# The evolutionary emergence of stochastic phenotype switching in bacteria

**DOI:** 10.1186/1475-2859-10-S1-S14

**Published:** 2011-08-30

**Authors:** Paul B Rainey, Hubertus JE Beaumont, Gayle C Ferguson, Jenna Gallie, Christian Kost, Eric Libby, Xue-Xian Zhang

**Affiliations:** 1 New Zealand Institute for Advanced Study and Allan Wilson Centre for Molecular Ecology & Evolution, Massey University at Albany, Auckland, New Zealand; 2 Max Planck Institute for Evolutionary Biology, Plön, Germany; 3 Delft University of Technology, Kavli Institute of Nanoscience, Department of Bionanoscience, Delft, The Netherlands; 4 Department of Biology, University of Washington, Seattle, USA; 5 Department of Bioorganic Chemistry, Max Planck Institute for Chemical Ecology, Jena, Germany

## Abstract

Stochastic phenotype switching – or bet hedging – is a pervasive feature of living systems and common in bacteria that experience fluctuating (unpredictable) environmental conditions. Under such conditions, the capacity to generate variable offspring spreads the risk of being maladapted in the present environment, against offspring likely to have some chance of survival in the future. While a rich subject for theoretical studies, little is known about the selective causes responsible for the evolutionary emergence of stochastic phenotype switching. Here we review recent work – both theoretical and experimental – that sheds light on ecological factors that favour switching types over non-switching types. Of particular relevance is an experiment that provided evidence for an adaptive origin of stochastic phenotype switching by subjecting bacterial populations to a selective regime that mimicked essential features of the host immune response. Central to the emergence of switching types was frequent imposition of ‘exclusion rules’ and ‘population bottlenecks’ – two complementary faces of frequency dependent selection. While features of the immune response, exclusion rules and bottlenecks are likely to operate in many natural environments. Together these factors define a set of selective conditions relevant to the evolution of stochastic switching, including antigenic variation and bacterial persistence.

## Introduction

The nature of information and its processing by living organisms is of longstanding interest [1-4]. Ability to acquire and process information is essential for expression of optimal phenotypic solutions [5]. In environments where information is reliable, sensory perception coupled with signal transduction systems allows organisms to readily tune their phenotype, or behaviour, to suit prevailing conditions [6]. However, in environments lacking useful information, or where changes in the nature of information are too rapid to process, then switching of phenotypes between alternate states via stochastic mechanisms – in essence, bet hedging – provides a viable alternative [4,7-9]. Although costly in the short term, stochastic phenotype switching provides an adaptive solution to life in the face of uncertainty [10]. Its adaptive value stems from the spreading of risk: the risk of being maladapted in the current environment being spread among variable offspring, each of which has some chance of surviving under future conditions [8].

The capacity to switch stochastically between heritable phenotypic states is common in the biological world [8,9,11], but especially so in bacteria. Observed initially as variation in the morphology of colonies arising from single bacterial clones [12], phenotypic switching has long been viewed as a property characteristic of bacterial pathogens [13]. However, advances in techniques for single-cell analysis show that stochastic switching is a near universal feature of living systems, which arises from fluctuations in transcription and translation, and affects the expression of numerous genes, regulatory networks and thus phenotypic states [14-20].

There are at least three instances in bacteria where the case for stochastic phenotype switching as adaptation has been argued. In the case of bacterial persistence, cells switch stochastically between growing and non-growing (persister) states. A combination of experiment and theory shows that stochastic switching can be adaptive in the face of periodic encounters with antibiotics despite the cost associated with non-growing cells [21-23]. A similar argument has been put forward to explain the competence to non-competence switch for natural DNA transformation in the soil bacterium *Bacillus subtilis*[17]. Like the persister state, competence is associated with periods of stagnation in an otherwise growing population and can be beneficial, despite the cost, provided the population periodically encounters conditions that kill growing cells [24]. In the case of persistence, demonstration that the optimal rate of switching is linked to the frequency of environmental change provides a compelling case for stochastic switching between growing and non-growing cells as adaptation tuned to the distribution of environmental fluctuations [23].

The third example stems from the study of obligate commensals -- and sometime pathogens -- of humans, such as *Haemophilus influenzae*, *Streptococcus pneumoniae*, *Neisseria meningitidis*, *Campylobacter jejuni*, and *Helicobacter pylori* (reviewed in [13,25]). For example, survival of *H. influenzae*, a major cause of meningitis, depends on avoidance of recognition by the host immune response. Given that moment-by-moment fluctuations in the state of the immune response cannot be predicted [26], *H. influenzae* survives by hedging its evolutionary bets. Central to this strategy are contingency loci: short repetitive DNA sequences that effect the expression of genes involved in critical interactions with the host. By virtue of their repetitive nature, contingency loci are prone to polymerase slippage: slippage causes localised hypermutation [27], which in turn causes heritable, stochastic switching of genes involved in commensal and pathogenic behaviour (antigenic variation). In conjunction with population growth, the capacity to stochastically switch means that highly polymorphic populations emerge rapidly from limiting and initially uniform inocula. The resulting phenotypic heterogeneity ensures that the risk of immune detection is spread among variable offspring, each of which has some chance of avoiding recognition.

While the molecular bases of contingency loci are well established (reviewed in [25]), the selective causes are unclear. In those instances where stochastic switching is a product of molecular noise, no evolutionary explanation is required (but this does not exclude the possibility that selection might exploit noise for adaptive ends). However, where the case for stochastic phenotype switching as an adaptation is strong, the selective causes are of considerable interest.

## Selective causes of stochastic phenotype switching

What ecological factors might promote the evolutionary emergence of stochastic phenotype switching? Fluctuating environmental conditions is a likely initial response [28]. While a sound response, ‘fluctuating environmental conditions’ does not define a precise set of ecological conditions. Indeed, the multi-dimensional nature of environmental heterogeneity [29], makes defining the appropriate set a considerable challenge.

Consider once again *H. influenzae*: during the course of colonising a new host, the bacterium faces fluctuating and unpredictable conditions, but the specific effects wrought by the immune response are numerous [30]. For example, in addition to unpredictable conditions, *H. influenzae* experiences environmental fluctuations with varying dynamics and degrees of uncertainty; whether or not bet hedging evolves – as opposed to environmental sensing – depends on various factors [10,23,31-35], including the existence and reliability of environmental cues [9,36,37], capacity of the population to respond by mutation and selection [10,38], the nature of the fitness landscape [33,39], and the cost-benefit balance of different strategies [23,31,33,38].

## Experimental studies

While many researchers have been intrigued by the challenge of explaining the evolution of stochastic phenotype switching (e.g., [23], [32], [39], [40]), the majority of studies have been theoretical and none readily account for how a switching genotype can arise *de novo* and increase in frequency in a population of non-switching types. From an experimental perspective, Moxon *et al*[13] outline a general strategy in which they envisage switching types arising from populations of non-switching bacteria when subjected to frequent changes in the selection pressures acting on particular gene products. Attempts by one of us to recreate this experiment met with failure because of difficulties – if not impossibilities – associated with identifying a set of reciprocal selection pressures that continuously select for contrasting changes at a single genetic locus. While selection for, say antibiotic resistance, results in a genetic change at a single locus, selection for sensitivity (where there is a sufficiently high fitness cost to allow selection for the evolution of sensitivity) typically results in subsequent mutational changes at an entirely different locus. In a recent experiment Freed *et al*[19] developed an elegant screen to identify *Salmonella* promoters displaying high levels of phenotypic noise by subjecting a library of fusions between chromosomal fragments of green fluorescent protein (GFP) to fluctuating selection. Although the authors identified intrinsically noisy promoters, they did not, at least over the course of the seven bouts of alternating selection, observe the de novo evolution of stochastic switching types. Quite possibly further selection would deliver the desired entities, but it is possible that ecological factors in addition to simple fluctuating selection are necessary.

As a gedanken experiment *H. influenzae* continues to be useful. There is little doubt that the bacterium experiences fluctuating selection as it encounters the host immune response, but fluctuating selection is likely to exert specific population effects that might also be relevant. For example, as *H. influenzae* populations increase in size, types not detected by the immune response stand a chance of becoming common: however, common types are likely to be detected and eliminated. At the moment of detection the population experiences strong frequency dependent selection: types that were common are eliminated and concomitantly the population collapses. Re-establishment of the population occurs via rare types that avoided immune detection.

**Figure 1:**
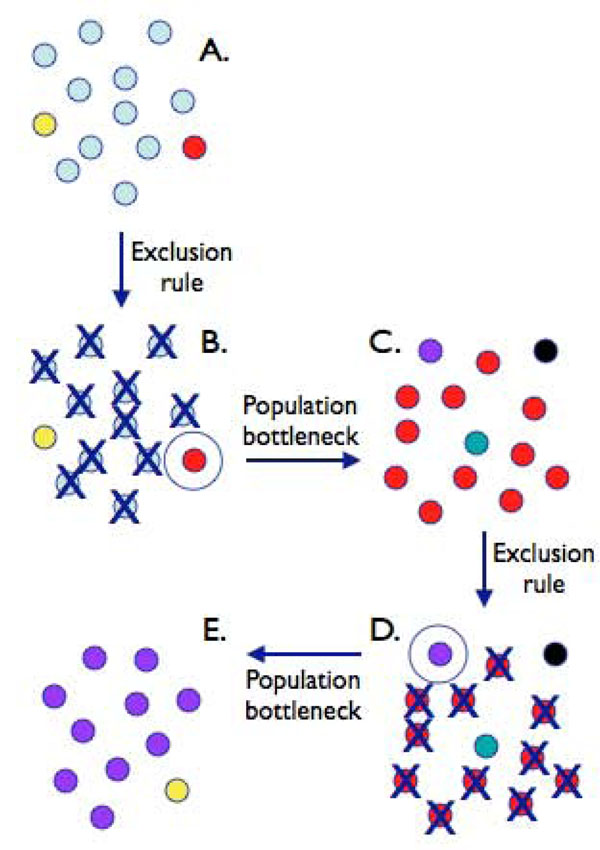
**Schematic representation of the dynamics experienced by a population evolving in the face of fluctuating selection wrought by the host immune response. A.) The population is founded by a single (blue) genotype.** During the course of growth, rare mutant types arise. B.) At some future moment the environment changes (e.g., the population is detected by the immune system) and common types are eliminated. C.) A single new (red) type avoids detection and proceeds to re-establish the population. A.-C.) The population experiences an ‘exclusion rule’ and passes through a bottleneck. The process is repeated: the red type becomes common, but is eventually detected and eliminated (D.). E.) The population once again passes through a single-cell bottleneck before being re-established from the rare (purple) type. In the face of such selective conditions a type that evolves the capacity to stochastically switch, at high frequency, between phenotypic states, has a clear selective advantage compared to a type that relies on spontaneous mutation to effect the change.

Recognition that bacteria such as *H. influenzae* experience these kinds of population effects was instrumental in the design of a recent experiment where populations of bacteria were allowed to evolve in the face of a selective regime that mimicked the dynamic fluctuations described above [41]. Specifically, populations of *Pseudomonas fluorescens* – a bacterium that does not undergo visible phenotypic switching—were subjected to strong frequency-dependent selection wrought by repeated imposition of an exclusion rule and bottleneck (Figure 1). Applied at the point of transfer between environments, the phenotype common in the current environment was assigned a fitness of zero and thus excluded from participating in the next round (the exclusion rule). In addition, also at the point of transfer, and so as to found the next bout of selection, a single phenotypically distinct type was selected at random from among the survivors (the bottleneck). In two of 12 replicate lines, stochastic switching types evolved after eight successive rounds of fluctuating selection—each punctuated by concomitant imposition of the exclusion rule and bottleneck.

## The *Pseudomonas* experiment

Details of the *Pseudomonas* ‘bet hedging’ experiment have been described previously [41]. Briefly, it involved repeated imposition of the exclusion rule and bottleneck on populations of *P. fluorescens* SBW25 transferred between static and shaken broth microcosms (Figure 1). Imposition of the exclusion rule and bottleneck was based entirely on colony morphology: twelve replicate static broth microcosms were founded by the ancestral genotype that produces smooth colonies on agar plates. After three days cells were diluted and plated. As is typical for such experiments [42] the resultant populations were highly polymorphic with respect to colony morphology. Smooth types were assigned a fitness of zero (they were excluded from all possibility of founding the next bout of selection) and a single colony of the numerically most dominant new type was chosen at random from the remaining colonies. The single colony type (one from each replicate microcosm) was then used to found the next set of microcosms that were then incubated under the aerated (shaking) regime. At three days cells were again diluted and plated: the type that founded the shaken microcosms was excluded and a single new type chosen to found the next bout of selection.

In two of the twelve lines, types that switched stochastically emerged at the ninth bout of selection. In both cases the switching genotypes produced colonies of two distinct types: opaque and translucent. As is characteristic of phenotype switching in pathogens [13], a colony of either type streaked across an agar plate gave rise to a mixed population of colonies. The switching phenotype was heritable and specific to just the colony morphologies of interest. Experiments that examined the mutation rate to traits such as phage and antibiotic resistance showed no evidence that the switching types were the consequence of a generalised mutator [41].

While stochastic switching was identified at the level of colony morphology, microscopic analysis showed the majority of cells from opaque colonies to be ensheathed in a thick capsule, whereas cells from translucent colonies were primarily devoid of capsules. Genetic analysis showed the capsule to be formed from a colanic acid polymer [41].

Additional genetic studies of one switching genotype unraveled a series of nine mutations – one responsible for each phenotypic shift – with the last being both necessary and sufficient to cause stochastic switching (see [41] for the list of mutations and [43] for details of Wsp, Aws and Mws). This last mutation, a single non-synonymous change in *carB* (C2020T (R674C)), was both necessary and sufficient to cause switching and did so when reconstructed in both the immediate ancestor of the switcher, and, surprisingly, in the ancestral type [41]. This finding showed that the phenotype caused by the *carB* mutation was not dependent on prior mutations for its phenotypic effects (no epistasis), however, measures of fitness revealed that spread of genotypes containing the *carB* mutation was dependent upon earlier mutations that rendered the immediate ancestor of the *car B* mutant less fit than the ancestral genotype SBW25 [41].

That a mutation in *carB* should generate stochastic switching was a considerable surprise. CarB is the large subunit of carbamoylphosphate synthase (CarAB, EC 6.3.5.5) and plays a pivotal role in the biosynthesis of pyrimidines and arginine. The R674C mutation is likely to decrease enzyme functionality leaving it necessary to explain how a decrease in the function of a key enzyme in central metabolism generates stochastic switching. The link is remarkably complex and convoluted, but resides in the connection between the pyrimidine biosynthetic pathway and the precursor for colanic acid production, UDP-glucose (J. Gallie, E. Libby, H. J. E. Beaumont and P. B. Rainey, unpublished). Full details will be published elsewhere, suffice to say that previous evidence that the switch is epigenetic [41] have now been established beyond doubt and involvea a metabolic feedback loop that generates bistable behaviour upon reduction in flux through the pyrimidine biosynthetic pathway (J. Gallie, E. Libby, H. J. E. Beaumont and P. B. Rainey, unpublished). Altogether this is a striking example of how natural selection can take advantage of molecular noise – in this case fluctuations in levels of intracellular metabolites – to generate adaptive solutions to survival in the face of uncertainty: the strategy minimises temporal variance precisely in accord with theoretical predictions [8,44].

## Theoretical studies show the broader significance of exclusion rules and population bottlenecks

That two stochastic switching genotypes arose *de novo*; that they increased from rare against a non-switching population, and did so in such a short period of time, is of special interest for those interested in the evolution of bet hedging [28,45]. The authors of the Beaumont *et al*[41] study argued that emergence of the switcher was attributable to the exclusion rule, which selected for phenotypic innovation, and population bottleneck, which negated the cost of bet hedging (the cost of producing types maladapted to the prevailing conditions). While evidence from the experiment is indeed suggestive, the conjecture is not proven. To perform the kinds of experiments necessary to explore the broader significance and robustness of the empiricists’ claims it would be necessary to carry out extensive experimentation on a scale that would be nigh impossible. Fortunately theoretical approaches provide a way forward.

Motivated by the experiment of Beaumont *et al*[41] Libby and Rainey [46] used a simple mathematical model to explore the competitive benefits of switching in populations subjected to repeated bouts of frequency-dependent selection imposed via exclusion rules and bottlenecks. They did so in order to assess the robustness and generality of the ecological conditions defined by the Beaumont *et al* experiment. Using mathematical and computer simulation models, the authors were able to show that even when initially rare, and when switching engenders a cost in Malthusian fitness, organisms with this capacity can invade non-switching populations and replace non-switching phenotypes. The simulations showed the results to be robust to alterations in switching rate, fidelity of the exclusion rule, bottleneck size, duration of the environmental state and growth rate.

One notable discovery arising from the mathematical simulations was recognition that the phenotypic states achieved by stochastic switching types are of secondary significance [46]. A selective regime involving strong frequency dependent selection – such as that experienced by *H. influenzae* when it encounters the host immune response – selects entities that generate phenotypic novelty: these entities can be successful despite poor ecological performance of the variant types. Survival stems from avoidance of recognition (being different), rather than generation of types fit to different states of the external environment. Again, in the context of pathogens confronted with the host immune system, the critical issue is to avoid detection. Being adapted to one environmental state versus another is of lesser significance, and where relevant, is likely the product of subsequent evolutionary refinement. Both the *Pseudomonas* experiment [41] and subsequent theory [46] emphasise stochastic switching as an adaptive response, not just to changes in the environment, but to change itself [7-9].

## Conclusion

Despite the apparent pervasiveness of stochastic phenotype switching, firm experimental evidence of bet hedging is remarkably scant [28,32] and the selective conditions for its evolutionary emergence essentially unexplored. A recent experiment with bacteria [41], combined with additional theory [46], show that ecological processes experienced by populations as they respond to fluctuating conditions, namely, exclusion rules and bottlenecks, are selective agents for stochastic switching, such that when initially rare, and when switching engenders a cost in fitness, organisms with this capacity can invade non-switching populations – and replace non-switching types.

Much remains to be discovered and the mechanistic detail is likely to prove important. Insight thus far indicates that population bottlenecks and exclusions rules might together be considered an ‘ecological recipe’ for the evolution of switching types. Indeed, it is not difficult to envisage the operation of such factors in many situations. For example, the arms race between phages and their bacterial hosts [47], the patchiness of nutrient sources [48], and therapeutic application of antibiotics [24] are likely to cause bacterial populations to experience population bottlenecks and exclusion rules of various types. Of particular relevance are the findings from theoretical work, which show exclusion rules and bottlenecks do not need to be stringently applied in order to favour stochastic phenotype switching [46].

Our molecular-level explorations of one of the switching types identified in the Beaumont *et al* experiment [41] show how selection can take advantage of molecular noise, but whether metabolic bistability is the starting point for the evolution of contingency loci [13] as evident in many bacterial pathogens remains unknown. There exist many exciting opportunities for future experimentation, on both the extant *Pseudomonas* switching types, but also for additional experiments that explore the subsequent evolution of these switchers, and investigate the response of other bacteria to similar selective conditions.

Given the role that stochastic switching has in the lives of many bacteria – bacterial persistence [22] being just one of many recently discovered examples – and the relevance of these behaviours to both medicine [e.g., 31] and industry [e.g., 49], there is need to consider the kinds of selective conditions that we as ‘manipulators’ impose on bacteria. While there may be circumstances where stochastic switching serves useful purposes – such as the design of genetic circuitry – there are likely to be many other situations where anthropogenic factors – such as the dose and timing of antibiotic therapies – could lead to the evolution of switching behaviours types with undesirable consequences.

## Competing interests

There are no competing interests.

## Authors' contributions

All authors contributed to this review drawing upon specific areas of expertise. All authors have read and approved the final manuscript.
